# Gelatinization, Retrogradation and Gel Properties of Wheat Starch–Wheat Bran Arabinoxylan Complexes

**DOI:** 10.3390/gels7040200

**Published:** 2021-11-05

**Authors:** Wenjia Yan, Lijun Yin, Minghao Zhang, Meng Zhang, Xin Jia

**Affiliations:** 1 Beijing Key Laboratory of Functional Food from Plant Resources, College of Food Science and Nutritional Engineering, China Agricultural University, Beijing 100083, China; sevenyan@cau.edu.cn (W.Y.); ljyin@cau.edu.cn (L.Y.); zmhao@cau.edu.cn (M.Z.); zhangmengcau@163.com (M.Z.); 2 Center of Food Colloids and Delivery for Functionality, College of Food Science and Nutritional Engineering, China Agricultural University, Beijing 100083, China

**Keywords:** arabinoxylan, wheat starch, gelatinization, retrogradation, gel properties

## Abstract

Gelatinization, retrogradation and gel properties of wheat starch–wheat bran arabinoxylan (WS–WBAX) complexes have been evaluated. The results of rapid viscosity analyzer (RVA), differential scanning calorimetry (DSC) and Fourier transform infrared spectroscopy (FTIR) confirmed that WBAX samples with larger Mw and branching degree (HWBAX) significantly impeded gelatinization process of starch by effectively reducing the amount of water available for starch gelatinization. DSC analysis showed that both molecular characteristics and additive amount of WBAX samples have an effect on the long-term retrogradation behavior of starch. For the rheological studies of WS–WBAX mixed gels, the elastic moduli (G’) and shear viscosity of WS–WBAX mixed gels increased with the increase in additive amount of WBAX. WS–HWBAX mixed gels exhibited the lower G’ compared with starch gels containing WBAX with lower Mw and branching degree (LWBAX) at the same amount. The scanning electron micrographs (SEM) revealed that the microstructures of WS–WBAX mixed gels were mainly affected by the amount of WBAX, but hardly by the molecular characteristics of WBAX. Texture profile analysis (TPA) showed that the cohesiveness of fresh WS–WBAX mixed gels became larger with an increase in the WBAX addition amount. The hardness of WS–WBAX mixed gels tended to increase over the 14-day storage.

## 1. Introduction

Starch is the main component in wheat, contributing to the characteristics of wheat-based foods that include moisture retention, viscosity, texture, taste and shelf-life [1]. The quality attributes of starch-containing products result from the specific gelatinization and retrogradation behavior of starch [2]. Gelatinization of starch occurs over the critical temperature in the presence of sufficient water; it is an irreversible phase transition process initiated by hydration and swelling of the amorphous region of starch [3]. Numerous studies have explored the effect of non-starch polysaccharides on the gelatinization behavior of starch. Ma et al. (2019) reported that Konjac glucomannan (KGM) addition raised the peak viscosity and breakdown values of corn starch [4]. Chen et al. (2016) suggested that the addition of xanthan and curdlan increased the peak viscosities [5] but decreased the final and setback viscosities of rice starch.

Retrogradation is a recrystallization process in which disaggregated amylose and amylopectin molecules in gelatinized starches reassociate to form ordered structures [6]. Starch retrogradation proceeds in two stages. The first phase of retrogradation (short-term retrogradation) occurs as the network formed between amylose molecules as paste cools down, forming a fresh elastic gel. Amylose retrogradation determines the initial hardness of a starch gel, and the stickiness and digestibility of processed foods. The second phase of retrogradation (long-term retrogradation) is associated with recrystallization of the outer branches of amylopectin [7]. The long-term retrogradation behavior of starch affects the long-term development of gel structure and crystallinity of processed starch [8]. Controlling retrogradation behavior and gel properties of starch has been crucial to control quality of starch-based products. Starch and non-starch polysaccharide complexes have been used in processed foods since about 1950 to overcome the shortcomings of native starches, for example, to protect starch granules against shear, improve product texture/rheology and hold moisture [9]. The effects of various non-starch polysaccharides with different molecular characteristics and additive amounts on starch-containing gels, such as xanthan gum, guar gum, gellan or carrageenan, have been studied extensively. Zhao et al. (2021) reported that soluble soybean polysaccharide (SSPS) has different anti-retrogradation effects on different types of starches [10]. The gel properties of starch gels, such as viscoelastic properties and textural properties, have been investigated in starch-hydrocolloids mixed gels. Luo et al. (2020) found that the storage modulus and hardness of Mesona chinensis polysaccharide (MCP)-starch mixed gels increased with the increasing of MCP concentration [11]. Xie et al. (2020) reported that the network structure formed between starch and TSP molecules enhanced the elastic properties of starch-containing gels [12].

Arabinoxylan (AX) is a non-starch polysaccharide isolated from cereal bran. It consists of a linear backbone of β-1,4-D-xylopyranose to which α-L-arabino furanose substituents are attached through O-2 and/or O-3 [13]. Additionally, arabinoxylan is considered a type of dietary fiber that has many health benefits, including a prebiotic effect and prevention of chronic disease [14]. Qiu et al. (2016) found that arabinoxylan with different additive amounts has little effect on gelatinization temperature of arabinoxylan–starch mixed systems [15]. Hou et al. (2020) showed that the long-term retrogradation degree of arabinoxylan–starch mixed gel decreased with increasing arabinoxylan due to the enhanced interaction between arabinoxylan and amylopectin [16]. Gudmundsson et al. (1991) claimed that arabinoxylan could retard starch gelatinization and increase the degree of retrogradation by reducing the water availability of starch [17]. The arabinoxylan–starch mixed gels retrograded for 7 days were slightly more elastic as compared with starch gels. These above studies showed that concentration, different sources of origin and varied molecular characteristics could have different impacts on gelatinization behavior, retrogradation behavior and gel properties of starch.

The objective of this study was to investigate the gelatinization, retrogradation behaviors and gel properties of wheat starch–wheat bran arabinoxylan (WBAX) mixed systems. In our study, the arabinoxylan with different molecular characteristics (Mw, arabinose to xylose ratio (A/X)) was obtained by alkaline extraction with graded ethanol precipitation. The gelatinization and retrogradation behavior of starch systems in the presence of the WBAX with different molecular characteristics and additive amounts were investigated using rapid viscosity analysis and thermal analysis. The gel properties of starch–arabinoxylan complexes were studied using scanning electron microscopic (SEM), rheological analysis and texture analysis. We hope to provide a theoretical basis for the functions of wheat bran arabinoxylan to control the physical properties of starchy foods.

## 2. Results and Discussion

### 2.1. Pasting Characteristics of Starch/WBAX Composite Systems

The RVA characteristics of wheat starch–wheat bran arabinoxylan with higher Mw and branching degree (WS–HWBAX) and wheat starch–wheat bran arabinoxylan with lower Mw and branching degree (WS–LWBAX) complexes are presented in Table 1. The values of peak viscosity and final viscosity increased with the increasing amount of WBAX in the system. High concentration WBAX could coat around the starch granules by forming hydration film, thus increasing the volume fraction of swollen starch granules and causing the increase of viscosity [18]. Similar findings were reported by Brennan et al. (2006) [19], in which the addition of Fenugreek gum increased the paste viscosity of wheat starch in a concentration-dependent manner. The peak viscosities and final viscosity of wheat starch with LWBAX were significantly higher than those with HWBAX at the same amount, respectively. Stronger interactions between LWBAX and swollen starch particles or leached amylopectin might be responsible for this result [20,21]. Funami et al. (2005) reported a similar trend in which the polysaccharides with lower branched degrees and Mw exhibited greater effect to increase the viscosity of the starch-containing system [22].

The pasting temperature of WS–WBAX complexes with 1% WBAX was significantly lower than those of WS systems in the presence of 0.25% of WBAX. This could be attributed to the increase in the effective concentration of starch in the continuous phase [23]. It was based on the mutual exclusion of each polymer due to thermodynamic incompatibility between wheat starch and WBAX [24]. Similar results were found in mixtures of potato starch and xanthan mixed system as reported by Conde-Petit et al. (1997) [25]. Increased effective concentration of starch enhanced the interactions between the starch granules, thus leading to gelatinization at a lower temperature [22]. Additionally, the interaction between WBAX and leached amylose could also be responsible for the reduction of pasting temperature of the system [21]. Compared with HWBAX at the level of 1%, LWBAX at the same additive account was more effective in lowering the pasting temperature. This suggested that the interaction between LWBAX and starch could be stronger. The breakdown value primarily reflects the stability of the wheat starch granules during the pasting process; a lowered breakdown value suggests the loss of granule integrity [26]. The higher the concentration of LWBAX, the higher breakdown values was observed. The breakdown values of WS–HWBAX complexes showed a similar trend. These results suggested that the starch granules became less resistant to thermal treatment and mechanical shearing with an increase in WBAX concentration [9]. Breakdown values of starch pastes containing the HWBAX were significantly lower than those of pastes containing LWBAX at the same amount, indicating that WS–HWBAX complexes exhibited higher stability during the pasting as compared with WS–LWBAX complexes. The lower breakdown value of WS–HWBAX might be caused by less swelling of the starch granules owing to the greater hydration capacity of HWBAX. HWBAX could also retard the amylose molecules in the amorphous regions. The amylose molecules would act as a diluent and an inhibitor of swelling to alleviate the breakdown of swollen starch granules [27,28].

Setback (SB) value indicates the short-term retrogradation degree of starch paste during the cooling process, especially the recrystallization and rearrangement of amylose molecules [29,30]. The setback values of WS–WBAX complexes increased with an increasing WBAX addition. Moreover, the setback values of starch pastes containing LWBAX were significantly higher than those of pastes containing HWBAX at the same amount. These results indicated that LWBAX exhibited stronger effects on promoting the retrogradation of starch during cooling as compared with HWBAX. It may be attributed to more leached amylose as described above.

### 2.2. Thermal Analysis

The onset temperature (T_o_), peak temperature (T_p_), conclusion temperature (T_c_) and endothermic enthalpy (ΔH_gel_), calculated from the DSC thermograms are listed in Table 2. WS–HWBAX showed retarded retrogradation as compared with wheat starch system containing LWBAX at the same account, represented by the larger values of T_o_, T_p_, T_c_. The ΔH_gel_ values of starch pastes containing the HWBAX were significantly lower than those of pastes containing LWBAX at the same amount, indicating WS–HWBAX complexes exhibited lower gelatinization degree. The lower gelatinization degree upon addition of HWBAX may be resulting from the limited water availability for starch gelatinization owing to the greater hydration capacity of HWBAX [9]. Significant differences in the polysaccharide hydration ability could be attributed to differences in chain architecture [31]. HWBAX molecules with larger Mw and branching degree could readily hydrate and consequently reduce water availability during gelatinization of wheat starch. Therefore, WS–HWBAX complexes had the lower gelatinization degree as compared to WS–LWBAX. This was in agreement with the results of RVA that WS–HWBAX complexes had less loss on the integrity of starch granules and lower breakdown values in comparison with WS–LWBAX complexes. Similar results have been reported by Luo et al. (2017) that inulin could enhance the gelatinization temperatures and reduce the enthalpies of gelatinization of wheat starch due to the higher hydration ability of inulin [26]. Interestingly, the thermal parameters (T_o_, T_p_ and T_c_) of WS–LWBAX complexes were independent of the additive amount of LWBAX, whereas there was a significant increase in T_o_, T_p_ and T_c_ when the HWBAX addition increased from 0.25% to 1%. In sum, the pasting properties of WS–WBAX complexes were affected by both the addition level and molecular characteristics of WBAX molecules.

### 2.3. FTIR

**Figure 1:**
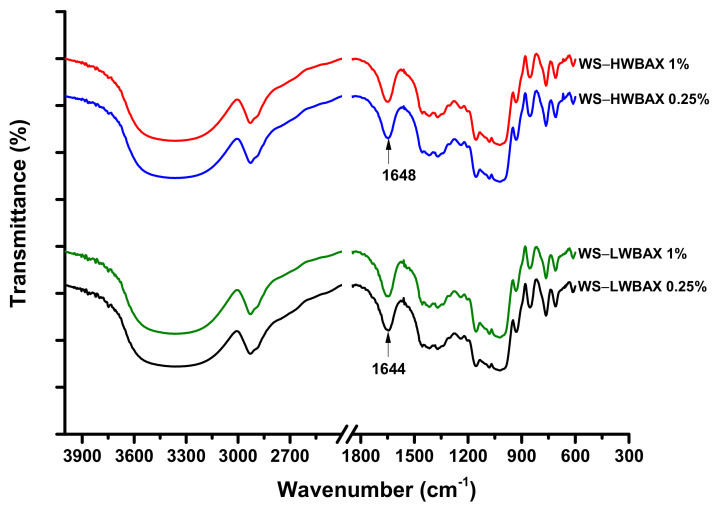
FTIR spectrums of WS–WBAX mixed pastes.

In order to explore the pasting mechanism of WS–WBAX complexes, the FTIR spectra of various WS–WBAX mixed pastes were detected and illustrated in Figure 1. The bands at 1080 and 1156 cm^−1^ are both attributed to the coupling of the C–O, C–C and O–H bond stretching, bending and asymmetric stretching of the C–O–C glycosidic bridge; these are associated with the ordered structures of the crystalline region [32,33]. There was no obvious difference in the relative peak intensities and peak position at 1080 and 1156 cm^−1^ among all samples. It indicated that no significant interaction between WBAX and the crystalline region of starch occurred during the pasting. For WS–LWBAX complexes, the band at 1644 cm^−1^, which is associated with hydrogen bonding within the hydroxyl group, is connected to content of adsorbed water molecules in the non-crystalline region of starch [34]. The wavenumber of maximum intensity at 1644 cm^−1^ shifted to 1648 cm^−1^ upon addition of HWBAX. HWBAX could exhibit higher hydration ability as compared with LWBAX samples due to their larger Mw and branching degree [31]. These results confirmed that the presence of the HWBAX weakened the affinity of the starches towards the water molecule due to its higher hydration, thus resulting in the lower gelatinization degrees as compared with WS–LWBAX complexes [35]. This result was consistent with the analysis of thermal properties of WS–WBAX complexes as described in Section 2.2. Similar trends have been reported by Krüger et al. (2003) that addition of more hydrophilic additives would result in lower heat transfer rates and water mobility, thus affecting the pasting properties of starch [36].

### 2.4. Steady Shear Rheological Properties

**Figure 2:**
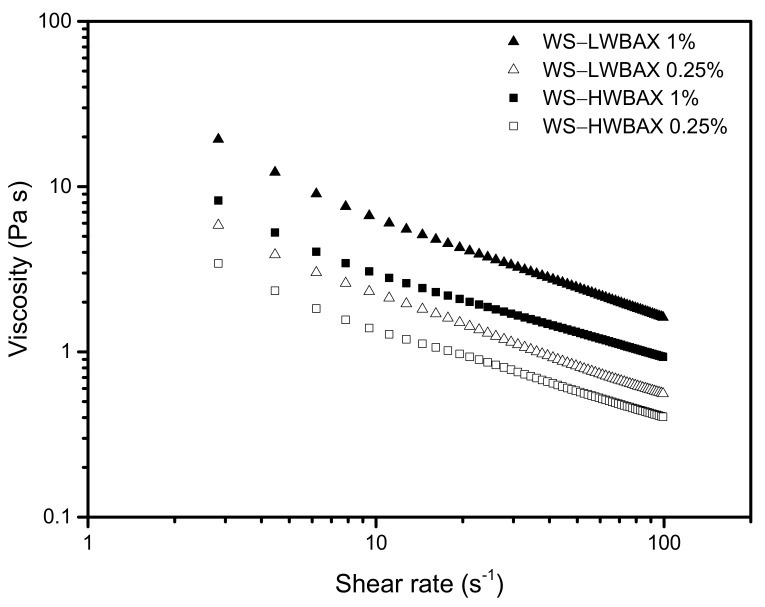
The apparent viscosity of WS–WBAX mixed fresh gels at 25 °C.

The steady shear rheological properties of starch–WBAX fresh gels at a shear rate of 0.01–100 s^−1^ was systematically studied as illustrated in Figure 2. The apparent viscosity of all samples decreased with increasing shear rate, indicating that WS–WBAX mixed gels exhibited non-Newtonian shear-thinning flow behavior. Similar results for other starch–polysaccharide systems were also reported [37,38]. The apparent viscosity of WS–WBAX mixed gels increased with the increase of WBAX from 0.25% to 1.0%. The starch pastes with HWBAX exhibited the lower viscosity as compared with starch paste containing the same amount of LWBAX at the same angular frequency. These results are also consistent with the final viscosity of the corresponding systems obtained during pasting. Singh et al. (2017) reported that the incorporation of gum arabic inhibited amylose leaching, leading to a decrease in the apparent viscosity [39].

### 2.5. Dynamic Rheological Properties

**Figure 3:**
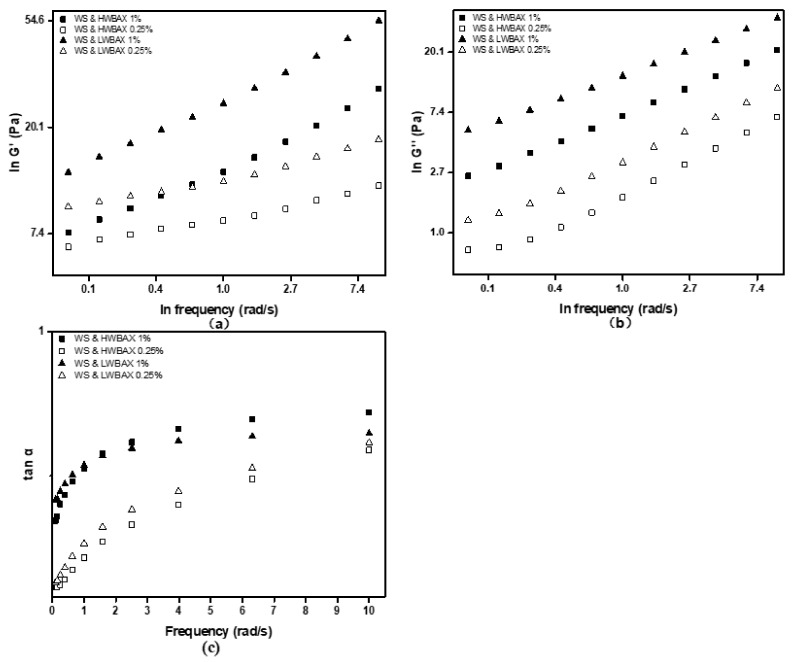
The storage modulus (G′) (**a**), loss modulus (G″) (**b**) and loss tangent (tanδ = G″/ G′) (**c**) of WS–WBAX mixed gels as functions of frequency.

The dynamic rheological behavior of wheat starch–WBAX mixed gels was analyzed at 25 °C. The storage modulus (G′), loss modulus (G″) and loss tangent (tanδ = G″/G′) of the gels as functions of frequency for WS–HWBAX and WS–LWBAX samples are presented in Figure 3. The tanδ values of all samples were less than 1 (Figure 3c), indicating the all starch–WBAX mixed pastes had been gelated and had an elastic solid-like behavior. The dynamic rheological data of ln G′ and ln G″ versus ln ω were subjected to linear regression as shown in Figure 3a,b. Fitting with a straight line, we were able to extract the slopes and intercepts as summarized in Table 3. G″ (slope = 0.411~0.539) showed much greater dependence on frequency than G′ (slope = 0.119~0.338), indicating that starch–WBAX mixed gels were weak gels [40,41]. The three-dimensional network of the starch paste developed during cooling was due to the formation of double helices between the amylose molecules [42].

The values of G′ for the starch–WBAX mixed gels increased with the increase in WBAX concentration, indicating that apparent gel strength of starch-containing pastes was enhanced by increasing the concentration of WBAXs. The starch gels with HWBAX exhibited the lower G′ as compared with starch paste containing the same amount of LWBAX at the same angular frequency. Liu et al. (2012) reported that before the recrystallization of starch gel occurred, the size of the crystalline lamellae in amylopectin cluster was observed to rise with increasing amylose content and the rigidity of starch granule structure was in proportion to its amylose content but in inverse proportion to the degree of granule swelling [9]. HWBAX inhibited the paste process, resulting in the less swelling power, and leached amylose of starch/HWBAX mixtures should explain why the G′ of WS–HWBAX was lower than that of WS–LWBAX in this study.

### 2.6. Microstructure of Gels

**Figure 4:**
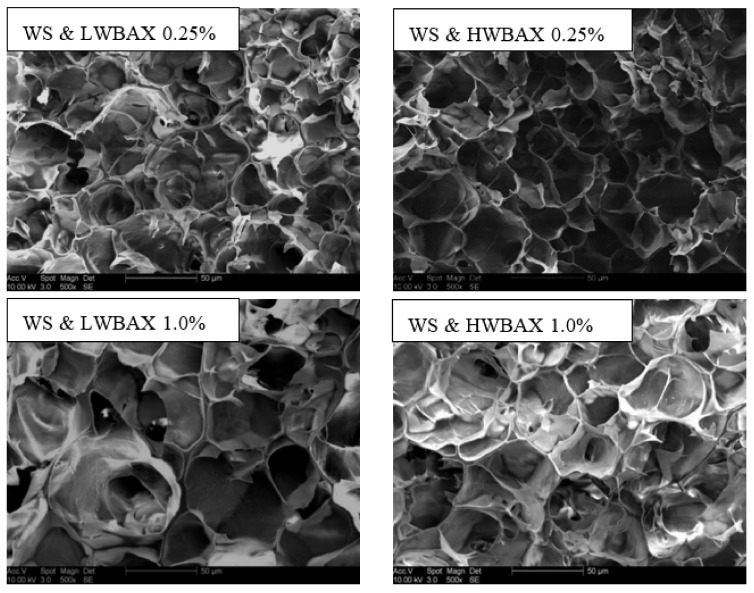
SEM images of WS–WBAX mixed gels.

The scanning electron micrographs (SEM) of cross-sections of different starch gels are shown in Figure 4. All starch-containing gels had a three-dimensional honeycomb-like network due to the swelling of granules and leaching of starch chains during heat treatment [43]. There was no obvious difference in the microstructure between the WS–HWBAX and WS–LWBAX mixed gel containing the same amount of WBAX. The wheat starch gels containing 0.25% of WBAX had small pores with thick cell walls, whereas the larger pore openings were formed in starch gels containing 1% of WBAX. The result of DSC analysis showed that the WS–WBAX complexes containing 1% WBAX exhibited the lower gelatinization degree as described in Section 2.2. Thus, there were not sufficient amylose chains in the amorphous region of the starch system to form a well-developed network when 1% WBAX was added. A similar result was reported by Hedayati and Niakousari (2018) [44], who found that the porosities of wheat starch-corn starch citrate (CSC) composites decreased with the increase in CSC level due to the limited granular swelling and gelatinization of starch–CSC composites.

### 2.7. The Degree of Long-Term Retrogradation

**Figure 5:**
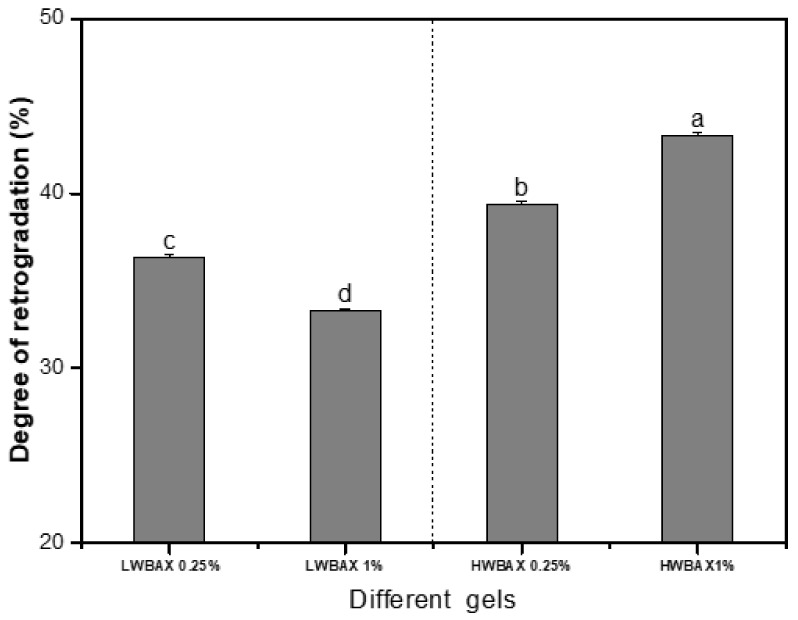
The degree of retrogradation for different WS–WBAX mixed gels. Different lowercase letters indicate significant differences among different mixed gels in the degree of retrogradation.

The retrogradation degree of gels decreased as the concentration of LWBAX increased, while the opposite trend was found for HWBAX (Figure 5). The degree of long-term retrogradation of LWBAX–starch gels was much lower than that of HWBAX–starch mixed gels, indicating that HWBAX–starch complexes exhibited a greater degree of starch retrogradation. Compared with HWBAX, LWBAX could exhibit stronger interactions with swollen starch particles or leached amylopectin [20,21]. This could retard the rearrangement of amylopectin and double-helical associations of amylose during refrigerated storage [8]. The above results confirmed the importance of molecular characteristics and the additive amount of WBAX samples on the long-term retrogradation properties of wheat starch gels.

### 2.8. Textural Attributes of Gels

**Figure 6:**
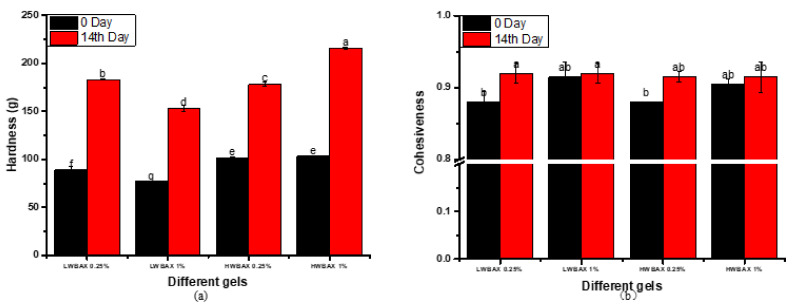
Hardness (**a**) and cohesiveness (**b**) of fresh and long-term retrogradated WS–WBAX gels. Different lowercase letters indicate significant differences among different mixed gels in the degree of retrogradation.

The textural properties of starch gel samples in terms of gel hardness and cohesiveness were measured on 0 day and 14 days during refrigerated storage (Figure 6). The hardness of starch gel in storage is highly correlated to retrogradation and can be used to evaluate the degree of retrogradation. It was observed that all the tested starch-containing gels hardened over the 14-day storage. The degree of hardening decreased with the increase in LWBAX, while HWBAX exhibited the opposite trend. This was similar to the trend of long-term retrogradation degree. The hardness of starch gels containing the LWBAX was significantly lower than that of gels containing HWBAX at the same amount.

Cohesiveness indicates the ability of a sample to withstand deformations. When the WBAX addition amount increased, the cohesiveness of the fresh starch-containing gels tended to be larger. The leaching of amylose out of the starch granules and their reassociation play the major role in formation of a cohesive starch gel. Starch gels with 1% of WBAX had lower gelatinization temperature and higher breakdown as compared with starch gels containing 0.25% of WBAX. Large quantities of amylose chains could leach out of the granules to form an interconnected network; therefore, the cohesiveness values of starch gel with 1% of WBAX were larger. Interestingly, there was no significant difference in cohesiveness of all WS–WBAX mixed gels retrograded for 14 days.

## 3. Conclusions

Both molecular characteristics and additive amounts of WBAX samples have an effect on the gelatinization, long-term retrogradation and gel properties of starch–WBAX complexes. HWBAX had a stronger inhibitory effect on the gelatinization process of starch as compared with LWBAX. LWBAX–WS complexes exhibited a lower long-term retrogradation degree than HWBAX-WS complexes. For fresh WS–WBAX mixed gels, the gel strength and shear viscosity of gels increased with increasing the amount of WBAX. WS–HWBAX mixed gels exhibited lower gel strength (G’) than starch gels containing LWBAX at the same amount. The microstructures of WS–WBAX mixed gels were mainly affected by the amount of WBAX, but hardly by the molecular characteristics of WBAX. The cohesiveness of WS–WBAX mixed gels became larger with an increase in the WBAX addition amounts. For WS–WBAX mixed gels retrograded at 4 °C for 14 days, both hardness and cohesiveness of WS–WBAX mixed gels tended to increase. Overall, results of the current study suggest that WBAX has great potential to modify the properties of starchy foods in the industry.

## 4. Materials and Methods

### 4.1. Materials

Commercial wheat starch (11.0% (*w*/*w*) moisture; 0.20% (*w*/*w*) ash content) was purchased from Shanghai Xiangben Industrial Co., LTD., China. The WBAX with lower Mw and branching degree (LWBAX, A/X = 0.45, Mw = 280 kDa, [η] = 4.13 cP), and WBAX with higher Mw and branching degree (HWEAX, A/X = 0.62, Mw = 754 kDa, [η] = 3.22 cP) were obtained through alkaline extraction with graded ethanol precipitation. Water was purified with a Milli-Q-Plus system (Millipore Corp., Billerica, MA, USA). Potassium bromide (KBr) was purchased from Aladdin Industrial Corporation (Shanghai, China).

### 4.2. Pasting Properties

Pasting viscosity characteristics of starch–WBAX complexes were determined using a Rapid Visco Analyzer (Newport Scientific Pty. Ltd., RVA-4, Sydney, Australia). Wheat starches (3 g, dry wt. basis) were added to an aluminum canister containing LWBAX or HWBAX solutions (0.25% and 1% (*w*/*w*)) to make a total weight of 28.0 g. The starch-WBAX suspension was run in the RVA instrument using the Standard Method 2 programmed in the Thermocline Software. Briefly, the suspensions were equilibrated at 50 °C for 1 min, and then raised to 95 °C within 9 min. They were maintained at 95 °C for 5 min, cooled to 50 °C at the same rate, held at 50 °C for 2 min. The rotation speed of the plastic paddle was at 960 rpm for the first 10 s, and then a lower constant speed (160 rpm) was used for the rest of the testing period. Each sample was run at least in duplicate. The Thermocline for Windows software was used to obtain pasting viscosity parameters (peak viscosity, breakdown, final viscosity, setback, peak time, pasting temperature).

### 4.3. Fourier Transform Infrared (FTIR) Analysis

The resulting starch–WBAX mixed pastes were immediately lyophilized and used for FTIR spectra measurements by an FTIR spectrometer (Perkin Elmer, spectrum 100). The samples were prepared in KBr tablet before measurement. The spectra were recorded from 600 to 4000 cm^−1^ and each sample was scanned 32 times with a resolution of 4 cm^–1^.

### 4.4. Differential Scanning Calorimetry (DSC)

The gelatinization and long-term retrogradation properties of starch/WBAX mixtures were measured using DSC (Shimadzu, TA-60 WS, Tokyo, Japan). Starch powder (2.5 ± 0.1 mg) was prepared with 7.5 μL of WBAX solutions in hermetically sealed aluminum pans and then equilibrated for 24 h at 4 °C. In the DSC measurements, the prepared pans were heated from 20 to 130 °C at a heating rate of 10 °C /min under a continuous flow of dry N_2_ gas, with an empty aluminum pan as a reference. The onset, peak and conclusion temperatures (T_o_, T_p_ and T_c_) of gelatinization and gelatinization enthalpy (H_gel_), were analyzed using Shimadzu TA-60 analysis software. For long-term retrogradation studies, these pans with the gelatinized samples were stored at 4 °C for 14 days before being re-scanned using the same temperature profile, and the retrogradation enthalpy (ΔH_ret_) was automatically evaluated. The retrogradation ratios (%) were calculated as the ratio of retrogradation enthalpy (∆H_ret_) to the gelatinization enthalpy (∆H_gel_).

### 4.5. Rheological Properties

The resulting arabinoxylan–starch mixed pastes were cooled to room temperature (25 °C), which took 30 min to obtain the fresh gels. The rheological properties of starch–WBAX fresh gels were determined under both steady and dynamic shear. The steady shear rheological properties were obtained with a rheometer (AR2000, TA Instruments, New Castle, DE, USA), using a parallel plate system (4 cm diameter) at a gap of 1000 μm. For steady shear measurements, the sample was loaded onto the plate of the rheometer and then sheared continuously from 1 to 100 s^−1^. Dynamic shear rheological properties were obtained from frequency sweeps over the range of 0.1–10 rad·s^−1^ at 1% strain. The 1% strain was in the linear viscoelastic region for each sample. TA rheometer data analysis software was used to obtain the experimental data and to calculate the apparent viscosity, the storage modulus (G′), loss modulus (G″) and loss tangent (tan*δ* = G″/G′). All rheological measurements were performed in triplicate.

### 4.6. Microstructure

For scanning electron microscopy (SEM) observation, the starch–arabinoxylan fresh gels sliced into thin pieces were freeze-dried after immersing in liquid nitrogen for 5 min. Lyophilized slices were attached to the SEM aluminum plate through the double-sided conductive carbon tape. After coating with gold, the microstructure of the cross-section of gels was observed under a scanning electron microscope (Hitachi SU8010, Tokyo, Japan) at an accelerating voltage of 20 kV.

### 4.7. TPA Analysis

Textural properties of the starch–arabinoxylan mixed gels after 0 day and 14 days storage were determined with a TA-XT plus texture analyzer (Stable Micro Systems, Surrey, UK). Each starch gel tested was cut from the center into cubes of 20 mm. Samples were compressed twice to 25% of original sample height using TA4/1000 probe at a pre-test, test, and post-test speed of 0.8 mm/s. The trigger type is ‘auto’ with trigger force of 5.0 g. Four texture profile analysis (TPA) parameters were calculated from the force/time curve: hardness and cohesiveness. Three replicate samples were tested and their average values with SD were calculated.

### 4.8. Statistical Analysis

The data obtained were expressed as mean ± standard deviation. Significant differences were between values at a α level of 0.05.

## Figures and Tables

**Table 1:** RVA characteristics for wheat starch–wheat bran arabinoxylan (WS–WBAX) complexes.

		Peak Viscosity (cP)	Breakdown (mPas)	Final Viscosity (mPas)	Setback (mPas)	Peak Time (s)	Pasting Temp (°C)
WS-LWBAX	0.25%	2916.33 ± 7.77 ^c^	836.00 ± 34.39 ^b^	4078.67 ± 29.09 ^c^	2004.00 ± 5.29 ^c^	10.60 ± 0.07 ^b^	87.62 ± 0.25 ^a^
	1.0%	5109.67 ± 17.62 ^a^	2266.00 ± 36.76 ^a^	5783.33 ± 34.96 ^a^	2929.67 ± 9.50 ^a^	10.37 ± 0.04 ^c^	67.84 ± 0.18 ^c^
WS-HWBAX	0.25%	2491.00 ± 37.24 ^d^	620.67 ± 1.15 ^c^	3621.67 ± 9.45 ^d^	1745.67 ± 24.91 ^d^	10.75 ± 0.11 ^b^	88.02 ± 0.23 ^a^
	1.0%	3706.00 ± 39.95 ^b^	782.33 ± 27.47 ^b^	5280.00 ± 65.34 ^b^	2358.00 ± 54.74 ^b^	11.39 ± 0.13 ^a^	86.75 ± 0.18 ^b^

^1^ Values shown are means ± standard deviations (n = 3). ^2^ Different letters indicate significate difference at the *p* < 0.05 level among all samples in a column.

**Table 2:** DSC measurements for pasting properties of WS–WBAX complexes.

	T_o_ (°C)	T_p_ (°C)	T_c_ (°C)	ΔH_gel_ (mJ/g)
WS-LWBAX 0.25%	57.24 ± 0.02 ^c^	62.76 ± 0.27 ^c^	67.79 ± 0.01 ^c^	9.00 ± 0.01 ^a^
WS-LWBAX 1.0%	57.39 ± 0.12 ^c^	62.89 ± 0.23 ^c^	67.63 ± 0.54 ^c^	8.30 ± 0.21 ^b^
WS-HWBAX 0.25%	58.47 ± 0.33 ^b^	63.95 ± 0.04 ^b^	69.29 ± 0.18 ^b^	8.34 ± 0.05 ^b^
WS-HWBAX 1.0%	59.74 ± 0.41 ^a^	64.73 ± 0.04 ^a^	69.86 ± 0.06 ^a^	7.44 ± 0.05 ^c^

^1^ T_o_, onset temperature; T_p_, peak temperature; T_c_, conclusion temperature; ΔH_gel_, enthalpy of gelatinization. ^2^ Mean values ± standard deviation of triplicates; values followed by the same letters in the same column do not differ significantly at *p* < 0.05 level.

**Table 3:** Slopes and intercepts of ln (G′, G″) versus ln ω (rad/s) data of different WS–WBAX gels at 25 °C.

Samples	G′			G″		
	Slope	Intercept	R^2^	Slope	Intercept	R^2^
WS–HBAX 1%	0.338	2.630	0.99	0.459	1.961	0.99
WS–HBAX 0.25%	0.119	2.149	0.99	0.539	0.643	0.99
WS–LWBAX 1%	0.3034	3.254	0.99	0.411	2.612	0.99
WS–LWBAX 0.25%	0.135	2.530	0.98	0.494	1.215	0.99

## References

[1] Wang S., Wang J., Yu J., Wang S. (2016). Effect of fatty acids on functional properties of normal wheat and waxy wheat starches: A structural basis. Food Chem..

[2] Copeland L., Blazek J., Salman H., Tang M.C. (2009). Form and functionality of starch. Food Hydrocoll..

[3] Annable P., Fitton M., Harris B., Phillips G., Williams P. (1994). Phase behaviour and rheology of mixed polymer systems containing starch. Food Hydrocoll..

[4] Ma S., Zhu P., Wang M. (2019). Effects of konjac glucomannan on pasting and rheological properties of corn starch. Food Hydrocoll..

[5] Chen T., Fang S., Zuo X., Liu Y. (2016). Effect of curdlan and xanthan polysaccharides on the pasting, rheological and thermal properties of rice starch. J. Food Sci. Technol..

[6] Ring S.G., Colonna P., I’Anson K.J., Kalichevsky M.T., Miles M., Morris V.J., Orford P.D. (1987). The gelation and crystallisation of amylopectin. Carbohydr. Res..

[7] Dobosz A., Sikora M., Krystyjan M., Tomasik P., Lach R., Borczak B., Berski W., Lukasiewicz M. (2019). Short-and long-term retrogradation of potato starches with varying amylose content. J. Sci. Food Agric..

[8] Wang S., Li C., Copeland L., Niu Q., Wang S. (2015). Starch Retrogradation: A Comprehensive Review. Compr. Rev. Food Sci. Food Saf..

[9] BeMiller J.N. (2011). Pasting, paste, and gel properties of starch–hydrocolloid complexes. Carbohydr. Polym..

[10] Zhao Q., Tian H., Chen L., Zeng M., Qin F., Wang Z., He Z., Chen J. (2021). Interactions between soluble soybean poly-saccharide and starch during the gelatinization and retrogradation: Effects of selected starch varieties. Food Hydrocoll..

[11] Luo Y., Shen M., Li E., Xiao Y., Wen H., Ren Y., Xie J. (2020). Effect of Mesona chinensis polysaccharide on pasting, rheo-logical and structural properties of corn starches varying in amylose contents. Carbohydr. Polym..

[12] Xie F., Zhang H., Xia Y., Ai L. (2020). Effects of tamarind seed polysaccharide on gelatinization, rheological, and structural properties of corn starch with different amylose/amylopectin ratios. Food Hydrocoll..

[13] Izydorczyk M., Biliaderis C., Bushuk W. (1990). Oxidative gelation studies of water-soluble pentosans from wheat. J. Cereal Sci..

[14] Mendis M., Simsek S. (2014). Arabinoxylans and human health. Food Hydrocoll..

[15] Qiu S., Yadav M.P., Liu Y., Chen H., Tatsumi E., Yin L. (2016). Effects of corn fiber gum with different molecular weights on the gelatinization behaviors of corn and wheat starch. Food Hydrocoll..

[16] Hou C., Zhao X., Tian M., Zhou Y., Yang R., Gu Z., Wang P. (2020). Impact of water extractable arabinoxylan with different molecular weight on the gelatinization and retrogradation behavior of wheat starch. Food Chem..

[17] Gudmundsson M., Eliasson A.-C., Bengtsson S., Aman P.P. (1991). The Effects of Water Soluble Arabinoxylan on Gelatinization and Retrogradation of Starch. Starch Starke.

[18] Liu S., Xiao Y., Shen M., Zhang X., Wang W., Xie J. (2019). Effect of sodium carbonate on the gelation, rheology, texture and structural properties of maize starch-Mesona chinensis polysaccharide gel. Food Hydrocoll..

[19] Brennan C.S., Suter M., Matia-Merino L., Luethi T., Ravindran G., Goh K., Ovortrup J. (2006). Gel and pasting behaviour of fenugreek-wheat starch and fenugreek–wheat flour complexes. Starch Starke.

[20] Rojas J., Rosell C., de Barber C.B. (1999). Pasting properties of different wheat flour-hydrocolloid systems. Food Hydrocoll..

[21] Shi X., BeMiller J.N. (2002). Effects of food gums on viscosities of starch suspensions during pasting. Carbohydr. Polym..

[22] Funami T., Kataoka Y., Omoto T., Goto Y., Asai I., Nishinari K. (2005). Effects of non-ionic polysaccharides on the gelatinization and retrogradation behavior of wheat starch. Food Hydrocoll..

[23] Alloncle M., Lefebvre J., Llamas G., Doublier J.L. (1989). A rheological characterization of cereal starch-galactomannan mixtures. Cereal Chem..

[24] Alloncle M., Doublier J.-L. (1991). Viscoelastic properties of maize starch/hydrocolloid pastes and gels. Food Hydrocoll..

[25] Conde-Petit B., Pfirter A., Escher F. (1997). Influence of xanthan on the rheological properties of aqueous starch-emulsifier systems. Food Hydrocoll..

[26] Luo D., Li Y., Xu B., Ren G., Li P., Li X., Han S., Liu J. (2017). Effects of inulin with different degree of polymerization on gelatinization and retrogradation of wheat starch. Food Chem..

[27] Lii C.Y., Tsai M.L., Tseng K.H. (1996). Effect of amylose content on the rheological property of rice starch. Cereal Chem..

[28] Qiu S., Yadav M.P., Chen H., Liu Y., Tatsumi E., Yin L. (2015). Effects of corn fiber gum (CFG) on the pasting and thermal behaviors of maize starch. Carbohydr. Polym..

[29] Pongsawatmanit R., Temsiripong T., Ikeda S., Nishinari K. (2006). Influence of tamarind seed xyloglucan on rheological properties and thermal stability of tapioca starch. J. Food Eng..

[30] Chen L., Ren F., Zhang Z., Tong Q., Rashed M.M.A. (2015). Effect of pullulan on the short-term and long-term retrogradation of rice starch. Carbohydr. Polym..

[31] Grossutti M., Dutcher J.R. (2016). Correlation Between Chain Architecture and Hydration Water Structure in Polysaccharides. Biomacromolecules.

[32] Higgins H.G., Stewart C.M., Harrington K.J. (1961). Infrared spectra of cellulose and related polysaccharides. J. Polym. Sci..

[33] Liu Q., Charlet G., Yelle S., Arul J. (2002). Phase transition in potato starch–water system I. Starch gelatinization at high moisture level. Food Res. Int..

[34] Sammon C., Bajwa G., Timmins P., Melia C.D. (2006). The application of attenuated total reflectance Fourier transform infrared spectroscopy to monitor the concentration and state of water in solutions of a thermally responsive cellulose ether during gelation. Polymer.

[35] Donmez D., Pinho L., Patel B., Desam P., Campanella O.H. (2021). Characterization of starch–water interactions and their effects on two key functional properties: Starch gelatinization and retrogradation. Curr. Opin. Food Sci..

[36] Krüger A., Ferrero C., Zaritzky N.E. (2003). Modelling corn starch swelling in batch systems: Effect of sucrose and hydrocol-loids. J. Food Eng..

[37] Chen L., Tong Q., Ren F., Zhu G. (2014). Pasting and rheological properties of rice starch as affected by pullulan. Int. J. Biol. Macromol..

[38] Ren Y., Jiang L., Wang W., Xiao Y., Liu S., Luo Y., Shen M., Xie J. (2020). Effects of Mesona chinensis Benth polysaccharide on physicochemical and rheological properties of sweet potato starch and its interactions. Food Hydrocoll..

[39] Singh A., Geveke D.J., Yadav M.P. (2017). Improvement of rheological, thermal and functional properties of tapioca starch by using gum arabic. LWT.

[40] Clark A.H., Dickinson E. (1991). Structural and mechanical properties of biopolymer gels. Food Polymers, Gels and Colloids.

[41] Rosalina I., Bhattacharya M. (2002). Dynamic rheological measurements and analysis of starch gels. Carbohydr. Polym..

[42] Goesaert H., Brijs K., Veraverbeke W., Courtin C., Gebruers K., Delcour J. (2005). Wheat flour constituents: How they impact bread quality, and how to impact their functionality. Trends Food Sci. Technol..

[43] Lee S.-W., Rhee C. (2007). Effect of heating condition and starch concentration on the structure and properties of freeze-dried rice starch paste. Food Res. Int..

[44] Hedayati S., Niakousari M. (2018). Microstructure, pasting and textural properties of wheat starch-corn starch citrate composites. Food Hydrocoll..

